# Prognostic Markers in Triple-Negative Breast Cancer (TNBC)

**DOI:** 10.3390/cancers18193181

**Published:** 2026-10-02

**Authors:** Ishaan Shah, Srushti Naik, Frank Arfuso, Sudha Warrier, Arun Dharmarajan, Sonal M. Manohar

**Affiliations:** 1 Department of Biological Sciences, Sunandan Divatia of School of Science, SVKM’s NMIMS (Deemed-to-be) University, Vile Parle (West), Mumbai 400056, India; 2 School of Human Sciences, The University of Western Australia, Perth, WA 6009, Australia; 3 Institute of Life Sciences, Atria University, Hebbal, Bangalore 560024, India; 4 Cuor Stem Cellutions Pvt. Ltd., Bangalore 562107, India; 5 Curtin Medical School, Curtin Health Innovation Research Institute, Perth, WA 6102, Australia; 6 Trichy SRM Medical College Hospital and Research Centre, Trichy 621105, India

**Keywords:** triple-negative breast cancer, molecular heterogeneity, prognosis, multi-marker panels

## Abstract

Triple-negative breast cancer (TNBC) is a highly aggressive and molecularly diverse subtype of breast cancer characterized by the absence of estrogen, progesterone, and human epithelial growth factor receptors. Despite advancements in therapeutic interventions, TNBC continues to remain associated with high metastatic potential, high likelihood of recurrence, and importantly, poor prognosis. Therefore, validated prognostic biomarkers are needed that can facilitate accurate prognostic assessment, drive treatment options, and lead to better clinical outcomes. This comprehensive review aims to provide a detailed overview of established and emerging prognostic biomarkers in TNBC, highlighting their biological relevance, clinical utility, and translational potential. We initially focus on the different classes of individual prognostic biomarkers and their limitations as far as prognostic stratification is concerned. We then move toward the development of multi-marker panels, further taking into account the critical factors limiting their clinical implementation. Development of standardized, clinically validated, and cost-effective multi-marker prognostic frameworks that can integrate the different aspects of tumor biology is the need of the hour. Such comprehensive approaches are expected to enhance prognostication and thereby improve outcomes in TNBC patients.

## Simple Summary

Triple-negative breast cancer (TNBC) is an aggressive and highly heterogeneous form of breast cancer marked by substantial risks of recurrence and metastasis. Unlike other subtypes of breast cancer, TNBC does not possess the three critical molecular traits that can act as key therapeutic targets for classification, necessitating the need for identification of reliable prognostic markers. However, the complex heterogeneity of TNBC has led to significant discrepancies in the prognostic value of individual biomarkers. This review summarizes the current landscape of individual prognostic biomarkers in TNBC and further emphasizes the transition toward promising multi-prognostic biomarker panels. We aim to highlight the need for such integrated prognostic approaches for effectively capturing the biological complexity of TNBC and thereby enabling more personalized therapeutic interventions.

## 1. Introduction

Breast cancer is widely implicated as the leading cause of cancer-related mortalities in women worldwide, accounting for a total of ~15.5% of cancer-associated deaths amongst women [1]. Breast cancer is a complex heterogeneous disease characterized by various distinct subtypes differing in their clinical and genomic patterns. Out of all of these subtypes, triple-negative breast cancer (TNBC) is responsible for 15–20% of all the breast cancer cases [2]. TNBC is particularly prevalent in women of younger age and those of American, African, and Hispanic ethnicity. Moreover, it is more common in women who have germline mutations in *BRCA1/2* or somatic mutation in *PTEN* or *TP53* [3]. The defining trait of TNBC is that it lacks the expression of three receptors, viz. estrogen receptors (ER), progesterone receptors (PR), and human epithelial growth factor receptor 2 (HER2). The absence of these receptors significantly limits the available treatment modalities, such as hormonal therapies or targeted therapeutic approaches, that otherwise prove to be effective in other breast cancer subtypes [4]. In clinical terms, TNBC is generally distinguished by an elevated mitotic index, higher histological grade, central necrosis, and extensive invasion of the lymphatic vessels (e.g., invasive breast carcinoma of no special type). These clinical features lead to the overall aggressive course of the disease and increase the risk of metastasis and subsequent recurrence [5]. Studies have reported that in metastatic TNBC, the median overall survival (OS) is between 9 to 13 months with conventional therapeutic interventions [6]. Consequently, accumulating scientific evidence exemplifies that the rates of recurrence are higher, especially within the first 3–5 years after diagnosis, making TNBC treatment extensively challenging [7]. In addition, the complex molecular heterogeneity of TNBC complicates its treatment choices. Notably, the triple-negative phenotype also encompasses rare special-type carcinomas that have low-grade morphology and relatively indolent behavior. These include salivary gland-type carcinomas, such as adenoid cystic carcinoma and secretory carcinoma, along with other rare subtypes, such as tall cell carcinoma with reversed polarity and adenosquamous carcinoma [7,8]. These subtypes typically exhibit more favorable outcomes in comparison to conventional high-grade TNBCs. Despite the recent advancements in the field of immune checkpoint inhibitors and poly ADP ribose polymerase (PARP) inhibitors that have enhanced outcomes in selected patient cohorts, efficient prognosis of TNBC continues to remain a crucial issue that needs to be addressed. As a result, there is an impending need to identify reliable prognostic markers that can effectively guide therapeutic decision-making and enable personalized management strategies.

Prognostic markers are nothing but biomarkers that provide detailed information about the progression of a particular disease and the damage done to the body by that disease. These markers provide key information about the tumor’s features, the grade of disease, and the overall impact on the patient’s body [9]. Thus, these markers offer insights into the natural course and expected clinical outcomes associated with the disease, irrespective of any treatment. By contrast, predictive biomarkers help to identify or predict whether a patient is likely to respond to a specific treatment regime and thereby derive benefits from the particular therapeutic intervention. As a result, predictive biomarkers primarily inform the process of treatment selection. It is important to note that these two categories of biomarkers are not mutually exclusive, as a biomarker may demonstrate both prognostic and predictive value depending on the clinical context and evidence available.

Traditional prognostic factors, including tumor size, lymph node status, and histological grade, offer important yet inadequate risk stratification in TNBC. Over the past few years, through the usage of high-throughput technologies and multi-omic approaches, the discovery of potential prognostic biomarkers in TNBC has been significantly expedited. In this comprehensive and critical review, we provide detailed insight into the different established as well as emerging prognostic biomarkers in TNBC, while highlighting their potential predictive implications wherever relevant. We also discuss the various issues pertaining to their clinical validation, existing limitations, and future perspectives for improving prognostic prediction in TNBC.

## 2. Methodology/Literature Search Strategy

A structured literature search was conducted to identify published evidence relevant to prognostic biomarkers and prognostic models in TNBC. The literature search was performed using PubMed, with additional relevant publications identified through manual screening of the reference lists of key articles and recent reviews. The search covered literature published from 2004 to 2026. Search terms were developed according to the major concepts addressed in this review and were combined using Boolean operators. The key search terms included were: “triple-negative breast cancer,” “prognosis,” “prognostic biomarkers,” “multi-markers,” and “multi-omics.” Additional biomarker specific terms such as “Ki-67,” “BRCA1/2,” and “TILs,” along with relevant individual genes and proteins, were incorporated wherever appropriate. Studies were considered eligible when they investigated prognostic associations, prognostic performance, or clinical relevance of biomarkers or biomarker-based models in patients with TNBC. Original research articles, reviews, systematic reviews, prospective or retrospective cohort studies, and clinical studies were included. Studies were excluded when they did not provide relevant information on TNBC, lacked prognostic or clinically relevant outcome data, or were unrelated to breast cancer. The identified literature was then screened for relevance based on the title and abstract, followed by assessment of the full text when necessary.

## 3. Molecular Heterogeneity of TNBC

Gene expression profiling allows TNBC to be categorized into several different molecular subtypes. Lehmann’s research group was the first to employ this approach, which subsequently led to the identification of six distinct molecular TNBC subtypes [10,11]:

- Basal-like 1 (BL1);
- Basal-like 2 (BL2);
- Immunomodulatory (IM);
- Mesenchymal (M);
- Mesenchymal stem-like (MSL);
- Luminal androgen receptor (LAR).

Apart from differing in their gene expression profiles, these TNBC molecular subtypes also vary in terms of potential therapeutic targets and signaling pathways. Table 1 provides an overview of the key molecular characteristics of these six TNBC molecular subtypes.

Such molecular subtyping provides valuable insights as far as prognostic stratification in TNBC is concerned. Research has suggested that patients with BL1 and IM subtypes have better survival rates and respond better to neoadjuvant chemotherapy in comparison to patients with BL2 and MSL subtypes [15]. Importantly, as mentioned in the above table, BL1 tumors are highly susceptible to chemotherapy, particularly platinum-based regimens, compared to BL2 tumors, which are often associated with inferior outcomes [5]. Additionally, the IM subtype has been linked to improved prognosis owing to its robust anti-tumor immune response as a result of extensive immune cell infiltration [16]. Conversely, the M and MSL subtypes are associated with a poor prognosis rate and decreased responsiveness to conventional chemotherapeutic approaches due to their association with the epithelial–mesenchymal transition (EMT) and stem cell-like characteristics [17].

Importantly, these six TNBC molecular subtypes were subsequently refined into four tumor-intrinsic subtypes encompassing the BL1, BL2, M, and LAR groups [15].

## 4. Individual Prognostic Markers in TNBC

In comparison to other types of breast cancer, TNBC is highly aggressive and has a rather dismal prognosis. As a result, it is essential to gain proper insight into the prognostic markers linked to TNBC to improve patient outcomes and drive therapeutic interventions [4].

Individual prognostic markers can, in turn, be categorized into different types as described in the following sub-sections.

### 4.1. Clinicopathological Prognostic Factors

Clinicopathological factors remain the cornerstone as far as initial prognostic assessment in TNBC is concerned. These factors are critical in clinical practice for evaluating the overall aggressiveness of the disease. The different types of clinicopathological factors include tumor size, histological grade, age at the time of diagnosis, lymph node involvement, and proliferation index. It is a well-known fact that larger TNBC tumors are associated with poorer OS. Histological grade is an indicator of tumor cell differentiation. As a result, higher histological grade TNBC tumors correlate with an increase in the aggressive nature of the disease, along with a worse prognosis rate [18]. Interestingly, research has indicated that younger patients typically exhibit more advanced stages of the illness, resulting in poorer outcomes [19]. Moreover, involvement of the lymph node is another strong predictor of prognosis in TNBC. Patients who have a positive lymph node status in TNBC tend to suffer from worse survival outcomes in comparison to their lymph-node-negative counterparts [20]. The expression of Ki-67 has also been linked to the proliferation of cancer cells, and elevated levels of Ki-67 in TNBC have been associated with poor prognosis. However, the reliability of Ki-67 as a prognostic biomarker is debatable due to lack of standardization and other methodologies [21]. Finally, histological tumor subtype is another clinicopathological factor that potentially may influence prognosis in TNBC. Unlike the Lehmann classification, which provides perspective into the underlying molecular heterogeneity, histological subtypes represent distinct morphological entities that may exhibit diverse clinical behaviors despite having similar receptor profiles [22]. A classic example of this is the favorable clinical outcomes observed in invasive breast carcinoma of no special type with medullary pattern, despite having a high Ki-67 index and high-grade morphology [23]. Similarly, as mentioned above, despite their high proliferative activity, rare histological subtypes like mucinous cystadenocarcinoma of the breast demonstrate a relatively favorable clinical outcome compared to conventional high-grade TNBCs [24]. Thus, histological subtype should also be considered alongside tumor size, grade, and other clinicopathological parameters when assessing prognosis in TNBC.

As a result, these clinicopathological markers remain fundamental for prognostic evaluation in TNBC. Nonetheless, they inherently suffer from certain limitations in terms of their ability to fully capture tumor heterogeneity and behavior, necessitating the need for integration with other biomarker types.

### 4.2. Genomic and Transcriptomic Markers

Genetic instability either due to mutations or because of alterations can significantly predispose an individual to cancer. Patients with genetic abnormalities in the *BRCA* genes, viz. *BRCA1* and *BRCA2*, are more likely to develop breast, prostate, and ovarian cancers [12]. *BRCA* is essential for homologous recombination-based DNA repair. Consequently, cells with mutations in *BRCA* lack the ability to repair double-stranded DNA breaks [5]. TNBCs with *BRCA* mutations possess a unique molecular profile that resembles the basal-like subtype [25]. Importantly, TNBCs in which *BRCA* is mutated hold key therapeutic significance as these tumors are more susceptible to targeted therapies such as PARP inhibitors and chemotherapy with platinum-based compounds [26].

Similarly, mutations in *TP53*, which encodes the p53 protein, are also commonly associated with most TNBC cases [12]. Several studies have revealed that p53 activation dramatically lowers the OS and disease-free survival (DFS) in TNBC patients and is linked to this aggressive form of breast cancer [27,28,29]. In another study, Chae et al. found that aberrant p53 protein expression correlated with a diminished response to chemotherapy in individuals suffering from TNBC [30]. Additionally, it has been reported that one-third of the TNBC patients express p63 or p73, which are other proteins belonging to the p53 family that potentially increase the sensitivity to platinum-based chemotherapy by multiple fold [31].

Another novel prognostic marker that can be useful in predicting patient outcomes in TNBC cases is ATP-binding cassette subfamily C member 9 (ABCC9). It plays an important role in TNBC as it is involved in cellular metabolism and clearance of drugs from cells. One of the major issues in TNBC is the resistance of cells to chemotherapy, and ABCC9 transporters help to remove chemotherapy drugs from cancer cells. Apart from imparting chemotherapeutic drug resistance, ABCC9 also helps cancer cells utilize energy and metastasize [32]. However, more investigation is required to understand the inhibitors and modulators of ABCC9 in combination with other therapies. Reducing the activity of ABCC9 can help make TNBC cells sensitive to chemotherapy and other treatment modalities. In the same study, Zhang et al. also found that NF-Kβ-activating protein-like (NKAPL) is significantly upregulated in TNBC [32]. NKAPL plays a significant role in cell survival and growth, and it is involved in promoting the NF-Kβ signaling cascade for processes such as inflammation and resistance to programmed cell death, which contribute to the aggressive nature of TNBC. NF-Kβ pathway activation confers resistance to apoptosis in TNBC cells, hence making it challenging to eliminate them from the body. NKAPL is also responsible for spreading cancer throughout the body. Hence, TNBC patients with higher levels of NKAPL have an increased incidence of metastasis and poor survival rates [32].

microRNAs (miRNAs) can also directly or indirectly impact the prognosis of TNBC, serving as potential biomarkers. Studies suggest that miR-374a-5p lowers the expression of arrestin beta 1 (ARRB1), which, in turn, is negatively associated with the histological grade of breast cancer but positively correlated with OS in TNBC cases [33]. Moreover, it was revealed that high expression of miR-449a was clearly linked to a better prognosis in TNBC [34]. Furthermore, Yao et al. discovered that TNBC patients with an elevated expression of miR-493 showed a superior DFS [35]. Qiu et al. even discovered that miR-3163 was associated with poor OS in androgen receptor (AR)-positive TNBCs, indicating its promising potential not only as a prognostic marker but also as a therapeutic target for such cases [36].

Apart from miRNAs, long non-coding RNAs (lncRNAs) have also been highlighted for their potential utility in assessing patient prognosis in clinical contexts. A study reported that aberrant expression of the lncRNA MALAT-1 is linked to a greater mortality rate in TNBC patients [37]. Similarly, LINK-A lncRNA has been associated with a lower recurrence-free survival in TNBC [38]. Interestingly, overexpression of the lncRNAs HULC and NEAT1 in TNBC patients has been correlated with poor OS [39,40,41]. Other notable lncRNAs associated with poor patient outcomes include HOTAIR, LOC101927359/AC105383, and LINC00393 [42,43,44]. On the other hand, certain lncRNAs have also been suggested to have a role in favorable prognosis [45]. For example, lncKLHDC7B inhibits cellular migration and invasion via induction of apoptosis, thereby reducing the aggressiveness of the IM subtype [46]. A novel breast-specific lncRNA—LINC00993—was found to bring about cell cycle arrest by targeting p14, p16, p21, and p53, resulting in tumor suppression [47]. Similarly, the lncRNA PTCSC3 suppresses the proliferation of TNBC cells by lowering lncRNA H19 expression [48]. Moreover, the lncRNA PTCSC3 even blocks TNBC by downregulating another lncRNA—MIR100HG [49]. Linc00921 sponges miR-9-5p, which causes an upregulation in the activity of leucine zipper tumor suppressor 2, in turn blocking tumorigenesis and EMT in TNBC [50].

Circular RNAs possess distinct characteristics and functions pertaining to cell proliferation and survival. The unique circular structure of circular RNAs makes them stable and resistant to degradation by the enzymes that target linear RNAs. Due to this stability, circular RNAs can be easily detected in various biological samples, such as blood and tissue [51]. It has also been reported that circular RNAs can act as promising prognostic markers in TNBC for predicting patient outcomes. Chen et al. highlighted the use of the circular RNA circEPSTI1 as a potential prognostic marker to detect TNBC. Upon performing micro assays, they found that the expression of circEPSTI1 was highly upregulated in TNBC compared to normal breast tissue. They also revealed that circEPSTI1 indirectly regulates the expression of *BCL11A*, a gene crucial in promoting cell proliferation and survival in TNBC. CircEPSTI1 works via a competitive endogenous RNA mechanism by acting as a sponge for certain microRNAs. CircEPSTI1 competes with the mRNA of BCL11A and binds to miR-4753 and miR-6809. Through this binding, circEPSTI1 sequesters them and prevents them from binding to the target mRNA. This sequestration ultimately results in the expression of *BCL11A*. The knockdown of circEPSTI1 has been shown to indirectly downregulate *BCL11A* expression, thereby blocking proliferation and inducing apoptosis [52]. Similarly, Yang et al. revealed that the expression of the circular RNA circPMSA1 is significantly elevated in TNBC. CircPMSA1 acts as a sponge for the microRNA miR-637, wherein it binds and inactivates it. The interaction of circPMSA1 and miR-637 triggers an increase in the level of Akt1, leading to the activation of the β-catenin signaling pathway, which ultimately influences the expression of cyclin D1 and thereby promotes cellular proliferation [53].

Thus, genomic and transcriptomic markers provide significant promise as far as prognostic stratification in TNBC is concerned.

### 4.3. Epigenetic Markers

Epigenetic alterations are pivotal in the development and progression of TNBC as they modulate gene expression without changing fundamental DNA sequences. Amongst the different epigenetic modifications, DNA methylation is the most extensively studied mechanism in TNBC. Stirzaker et al. reported that TNBC patients with lower methylation profiles exhibited a superior rate of survival in comparison to those with high methylation profiles [54]. Clinically, hypermethylation of *BRCA1* serves as a negative prognostic marker, causing a reduction in the OS and DFS of TNBC patients [55]. Apart from *BRCA1*, hypermethylation of other genes, viz. *FOXA1*, *GATA3*, and *TMS1*, can also contribute to the initiation of breast cancer [56,57,58]. Studies have even reported that several important genes involved in breast cancer, such as *SOX2* and *HIF-1α*, can be suppressed upon hypermethylation via DNA methyltransferase 3α (DNMT3A), leading to poor prognosis [59,60]. On the other hand, hypomethylation of *VWCE*, *TSPAN9*, and *ADAM12* promotes the proliferation and migration of TNBC cells and is thereby associated with poor outcomes [61,62]. Similarly, hypomethylation of *ST8SIA1*, which encodes ganglioside D3 synthase enzyme, has been correlated with an unfavorable relapse-free survival and lower OS in individuals suffering from breast cancer [63].

Histone post-translational modifications, which include methylation, acetylation, phosphorylation, and ubiquitination, to name a few, form another crucial component of epigenetic regulation in TNBC. Elevated levels of the methyltransferase enzyme enhancer of zeste homolog 2 (EZH2) have been reported to correlate with enhanced proliferation of tumor cells in TNBC [64]. EZH2 also hyperactivates the expression of pyruvate kinase M2 (PKM2) enzyme, resulting in disruption of carnitine metabolism and blockage of TNBC cell plasticity, ultimately exacerbating poor overall prognosis [65]. Additionally, in a retrospective analysis of 159 TNBC patients, the histone methyltransferase hSETD1A was linked to a lower survival rate along with an increase in the overall aggressiveness of the disease [66]. Besides methylation, histone acetylation has also been widely regarded to have implications in the progression of breast cancer. HDAC8, a histone deacetylase, is highly prevalent in breast cancer tissues and is responsible for the dissemination and EMT of breast cancer cells, serving as a negative biomarker for prognosis [67]. Another histone deacetylase—HDAC3—has been revealed to be significantly hyperactivated in TNBC cell lines in comparison to other luminal cell lines [68].

Hence, epigenetic biomarkers can offer valuable insights pertaining to the regulatory processes occurring in TNBC and subsequently aid in efficient prognostic assessment.

### 4.4. Protein-Based Markers

Protein-based biomarkers serve as one of the most clinically accessible tools for prognostic stratification in TNBC. These markers represent the direct functional state of tumor cells and the associated microenvironment, providing a comprehensive understanding of the critical cellular phenomenon. Amongst the different protein-based biomarkers, AR (belonging to the steroid receptor family of proteins) is commonly expressed in the majority (70–90%) of breast cancer cases [69,70,71]. Elevated expression of AR is observed in ~10–50% of TNBC patients [3]. Interestingly, TNBCs in which AR is positively expressed have been linked to a diminished proliferative activity, lower disease stage, and histological grade compared to AR-negative TNBCs [72,73]. Additionally, studies have also revealed that AR expression coincides with improved DFS and OS in TNBC [70,71]. Therefore, the absence of AR expression can be potentially correlated with higher rates of tumor recurrence and metastasis.

Sukumar et al. demonstrated that epidermal growth factor receptor (EGFR) is frequently overexpressed in TNBC in comparison to the other molecular subtypes, and Rakha et al. reported that the presence of EGFR in TNBC is associated with an unfavorable response to chemotherapy [74,75]. As a result, several therapeutic approaches involving the use of EGFR tyrosine inhibitors or anti-EGFR monoclonal antibodies are currently being investigated either singly or in combination with chemotherapy [76,77,78].

Vascular endothelial growth factor (VEGF) is found in ~30–60% of all TNBC cases [79]. According to a study by Linderholm et al., levels of intra-tumoral VEGF were found to be considerably greater in TNBC patients in comparison to non-TNBC ones [80]. Importantly, the monoclonal antibody bevacizumab, which specifically binds to VEGF-A, has been considered as a therapeutic alternative for TNBC treatment [81]. Some studies have suggested that fibroblast growth factor is also correlated with an inferior OS in TNBC [82,83].

Moreover, the expression of programmed death-ligand 1 (PD-L1), commonly present on the surface of tumor cells, has also been observed in ~20% of TNBC cases [84]. PD-L1 expression has been associated with an increased OS, metastatic-free survival, and complete pathological response rate in TNBC [84,85]. Hence, the administration of monoclonal antibodies against PD-L1 (e.g., atezolizumab) as well as programmed cell death protein 1 (e.g., pembrolizumab) has been recommended as a part of immunotherapy to curb TNBC [86,87].

Additionally, the inactivation of E-cadherin has been associated with adverse prognosis in TNBC. Apart from contributing to cell adhesion, it also plays a role in cellular proliferation and migration, and it acts as a metastasis suppressor. Loss of E-cadherin disrupts cell-to-cell adhesion, leading to EMT, which, in turn, is responsible for increased invasiveness and metastatic potential [88]. Interestingly, even aquaporins have been implicated in influencing cellular proliferation and survival in TNBC. Zhu et al. demonstrated that the overexpression of aquaporins-3 and -5 was critically linked to vital parameters such as tumor size, lymph node status, and metastasis [89]. They also highlighted the involvement of aquaporin-5 in the EMT of breast cancer cells through activation of the Wnt/β catenin signaling pathway. Furthermore, aquaporin-5 upregulates the expression of *BCL-2*, an anti-apoptotic gene, and downregulates pro-apoptotic genes such as *Caspase-3*, further contributing to tumor progression [90].

Therefore, protein-based biomarkers play a key role in bridging the gap between tumor biology and its clinical applications in TNBC.

### 4.5. Immune and Tumor Microenvironment-Related Markers

The spatial organization of immune components in conjunction with the composition of microenvironmental elements significantly impacts tumor progression, metastatic activity, and therapeutic response. As a result, numerous immune and tumor microenvironment-related biomarkers have surfaced as valuable prognostic indicators in TNBC.

Tumor-infiltrating lymphocytes (TILs), which form a part of the adaptive immune system involved in the tumor microenvironment, are extensively validated biomarkers in TNBC. The expression of TILs is strongly upregulated in about 20% of all TNBC cases [74]. TILs are generally present within the tumor (intra-tumorally) or around the adjacent tissue stroma, allowing them to serve as effective prognostic indicators. Upon investigation of tumor samples across several clinical studies, it was found that an increase in the number of TILs correlated with an enhanced OS, DFS, and pathological response rate following neoadjuvant chemotherapy in early TNBC cases [91,92]. Another extensive clinical study demonstrated that elevated expression of TILs was linked to marked improvement in the overall response rate of metastatic TNBC patients to pembrolizumab monotherapy [93]. A study along similar lines also revealed that the presence of higher TILs was associated with better clinical outcomes with pembrolizumab monotherapy compared to single-agent chemotherapy in patients with previously treated metastatic TNBC [94]. Because of this compelling data, TIL evaluation is being progressively integrated into clinical and translational research in TNBC.

Recently, immune-related genes have been explored for their promising potential in prognostic stratification in TNBC. Marchetti et al. used a detailed bioinformatic approach to identify immune-related genes positively associated with DFS in TNBC patients [95]. Among these genes, *IL18R1* is mainly involved in the activation of different lymphocytes and exhibits specific expression patterns related to survival predictions. Different levels of this potential prognostic biomarker in immune and TNBC cells revealed that there are significant variations between different tumors, which, in turn, could be useful in predicting patient outcomes. The findings from their study have significant implications as this biomarker may aid in creating immune-focused treatment or may act as an indicator of treatment response. Apart from *IL18R1*, other immune-related genes identified by Marchetti et al. that can act as potential prognostic markers for TNBC include *CD53*, *TRIM*, *Jaw1*, *LTB*, and *PTPRCAP*.

Inositol 1,4,5-trisphosphate 3-kinase C (ITPKC) is primarily known for its negative role in T-cell activation, and its reduced function is associated with Kawasaki disease. Interestingly, Oshi et al. found that ITPKC can act as an independent prognostic factor in TNBC [96]. High levels of ITPKC were linked to worse survival in TNBC, whereas tumors that had low ITPKC expression were associated with a better pathological response rate post neoadjuvant chemotherapy. Additionally, it was even revealed that high infiltration of tumor-associated macrophages is correlated with an increase in aggressiveness as well as unfavorable prognosis in TNBC patients after neoadjuvant chemotherapy [97].

Immune and tumor microenvironment-related biomarkers represent one of the most promising areas of prognostic research in TNBC. Among these, TILs have demonstrated consistent therapeutic significance, while further research is needed to identify other markers to match the growing interest in immunotherapy for TNBC.

### 4.6. Circulating and Liquid Biopsy-Based Markers

Tissue biopsy remains one of the most standard and reliable methods for clinical diagnosis of any tumor. It helps to determine the histology and stage of the tumor. However, it suffers from a few limitations in terms of its invasive nature and limited potential for assessing the spatial and temporal heterogeneity of tumors [98]. In such scenarios, liquid biopsy has emerged as a promising alternative approach to study tumor biology. Liquid biopsy involves the detection of tumor-derived components in body fluids such as blood, urine, and cerebrospinal fluid. Its major advantages are that it is a non-invasive approach, it allows efficient evaluation of tumor heterogeneity, and it enables real-time monitoring of tumor burden and progression [99]. In addition, it is particularly beneficial for early detection of cancer in asymptomatic individuals and those at high risk, potentially improving survival rates [98].

Tumor cells that separate from the main tumor and subsequently enter the bloodstream are known as circulating tumor cells (CTCs). CTCs can be detected through several methods, such as flow cytometry, immunohistochemistry, and RT-PCR [100]. They help to elaborate the genetic and phenotypic heterogeneity of primary tumors as well as metastatic lesions, thereby serving as useful prognostic indicators [99]. Reports suggest that CTCs can offer valuable insights as far as early detection of TNBC is concerned. Testing for CTCs can help to identify disease recurrence prior to emergence of clinical symptoms, thereby facilitating a quick and pre-emptive therapeutic response amongst patients [98]. According to a recent study by Chen et al., higher baseline CTC counts were found to be negatively correlated with OS and progression-free survival in TNBC cases [101]. Another factor that further propagates the use of CTCs as critical prognostic tools is their easy extraction from peripheral blood, enabling the development of refined treatment plans. Additionally, single-cell technologies in the form of single-cell RNA sequencing and single-cell proteomics can substantially enhance the detection sensitivity as well as specificity of CTCs by providing key information pertaining to resistant subpopulations, metastatic spread, and tumor heterogeneity [102,103].

Apart from CTCs, circulating cell-free DNA (cfDNA) is also frequently released into the bloodstream via active secretion by cells or as a part of cell death mechanisms such as apoptosis and necrosis. Studies suggest that the cfDNA concentration is usually on the higher side in advanced malignancies compared to early-stage patients or healthy individuals [99]. cfDNA comprises tumor-derived DNA as well as non-tumor DNA. Tumor-derived DNA makes up only a tiny fraction of the total cfDNA and is specifically known as circulating tumor DNA (ctDNA). Both cfDNA and ctDNA are being extensively explored for their prognostic value in TNBC. Accumulating scientific evidence indicates that cfDNA and ctDNA levels are substantially correlated with OS and progression-free survival in breast cancer, especially in metastatic tumors [104]. Importantly, since the presence of ctDNA can only be detected through the identification of genetic mutations pertaining to cancer, it has the potential to not only monitor disease progression but also aid in distinguishing malignant breast cancer conditions from benign ones [105]. Moreover, ctDNA accurately represents the current tumor burden (owing to its short half-life) and has also proven to be useful in determining minimal residual disease, thereby serving as a promising investigational marker for prognosis as well as treatment in TNBC [99,106]. As a result, detection of ctDNA is a more specific approach since it considers tumor alterations. Nevertheless, its detection is more challenging and costly. cfDNA, on the other hand, has predictive significance but lacks the desired genetic specificity compared to ctDNA [107].

Interestingly, extracellular vesicles (EVs) derived from tumors also encompass DNA, proteins, miRNA, and lipids that mirror a tumor’s pathophysiological condition, including TNBC. Circulating exosomes, a type of EV, serve as one of the key tools as far as liquid biopsy approaches are concerned. Exosomes possess exceptional stability across a range of body fluids, viz. blood, saliva, and urine [108]. Second, the range of proteins and genetic material within exosomes potentially enables the analysis of several targets from a single sample itself [109]. Li et al. reported that the expression of CD151, responsible for the migration and invasion of TNBC cells, is elevated in serum exosomes derived from TNBC patients, implying its potential utility as a prognostic TNBC biomarker [110]. Similarly, EVs derived from TNBC cells and plasma have been found to express deubiquitinase UCHL1 in substantially higher amounts, ultimately leading to the extravasation of breast cancer cells [111]. EVs loaded with miR-223 are also regarded as prognostic indicators in TNBC. TNBC-associated macrophages release miR-223, which, in turn, promotes breast cancer invasion [112]. In addition, TNBC EVs frequently exhibit increased expression of miR-10b, which targets HOXD10 and KLF4 to make non-malignant cells more invasive [113]. Lastly, EVs containing miR-105 are often regarded as biomarkers in early-stage TNBC [114].

**Figure 1:**
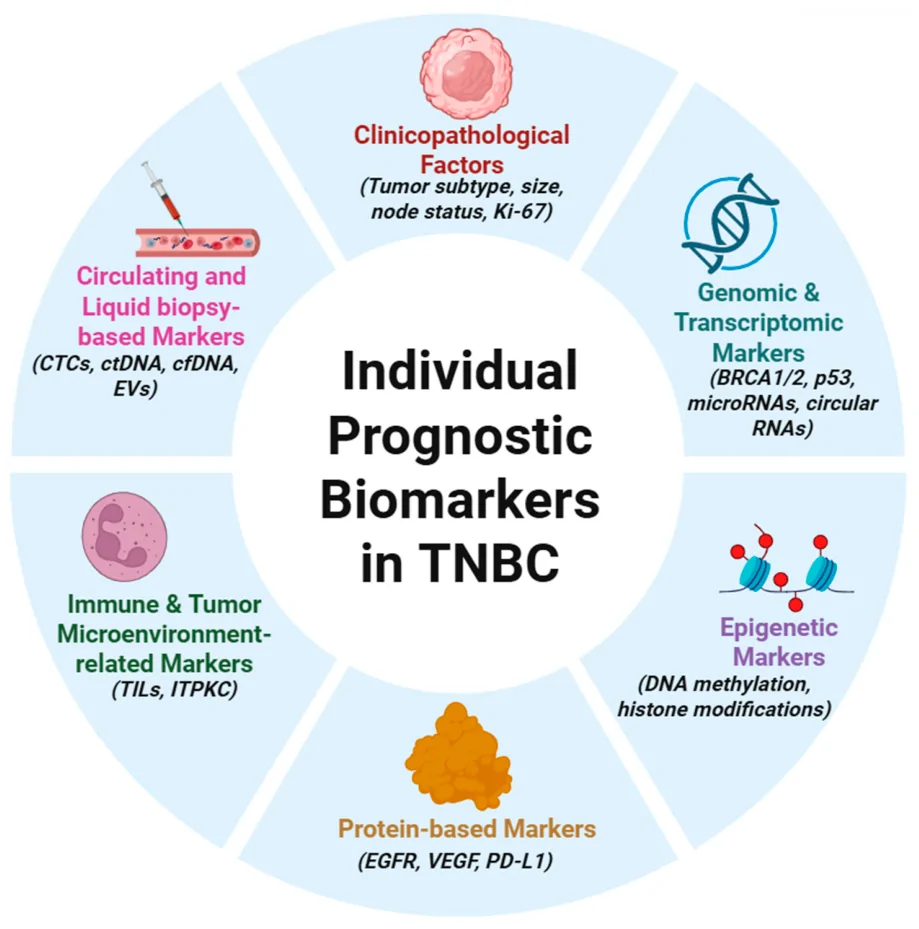
Classification of individual prognostic biomarkers in TNBC.

Figure 1 provides an overview of the different classes of individual prognostic markers in TNBC.

## 5. Limitations of Individual Prognostic Biomarkers in TNBC Prognostication

Despite the emergence of numerous prognostic biomarkers in TNBC, it is quite challenging and difficult to only rely on a single biomarker class for prognostic stratification. As mentioned previously, TNBC is a molecularly diverse disease and involves a complex interplay between genetic, epigenetic, immune, and microenvironmental factors. An individual prognostic biomarker in isolation fails to fully capture the complex heterogeneity of TNBC tumors. The overall performance of any biomarker can be greatly impacted by intra-tumoral and inter-tumoral heterogeneity, causing significant variations in its prognostic value among patient cohorts and molecular subtypes [115]. For example, immunological markers are extremely significant in the IM subtype, but they are irrelevant as far as the LAR subtype is concerned. Moreover, the expression of individual biomarkers may fluctuate dynamically throughout the disease course and in response to therapeutic interventions (a phenomenon known as temporal heterogeneity). As a result, a biomarker that suggests a positive prognosis at the time of diagnosis may entirely reverse its prognostic significance following treatment. In addition, differences in terms of assay variability, scoring procedures, and cut-off criteria further exacerbate the issues pertaining to therapeutic decision-making [116]. Lastly, despite the fact that several categories of individual prognostic biomarkers have been developed, very few actually provide validated information pertaining to prognosis. Most of the above mentioned biomarker classes are investigational in nature, requiring prospective and external validation prior to their clinical implementation. Table 2 divides the individual prognostic biomarkers into different categories based on their clinical implementation for TNBC.

Therefore, it is practically impossible for a single prognostic biomarker to define the entire clinical trajectory of TNBC.

Table 3 further illustrates the challenges and limitations of each prognostic biomarker type in detail.

**Figure 2:**
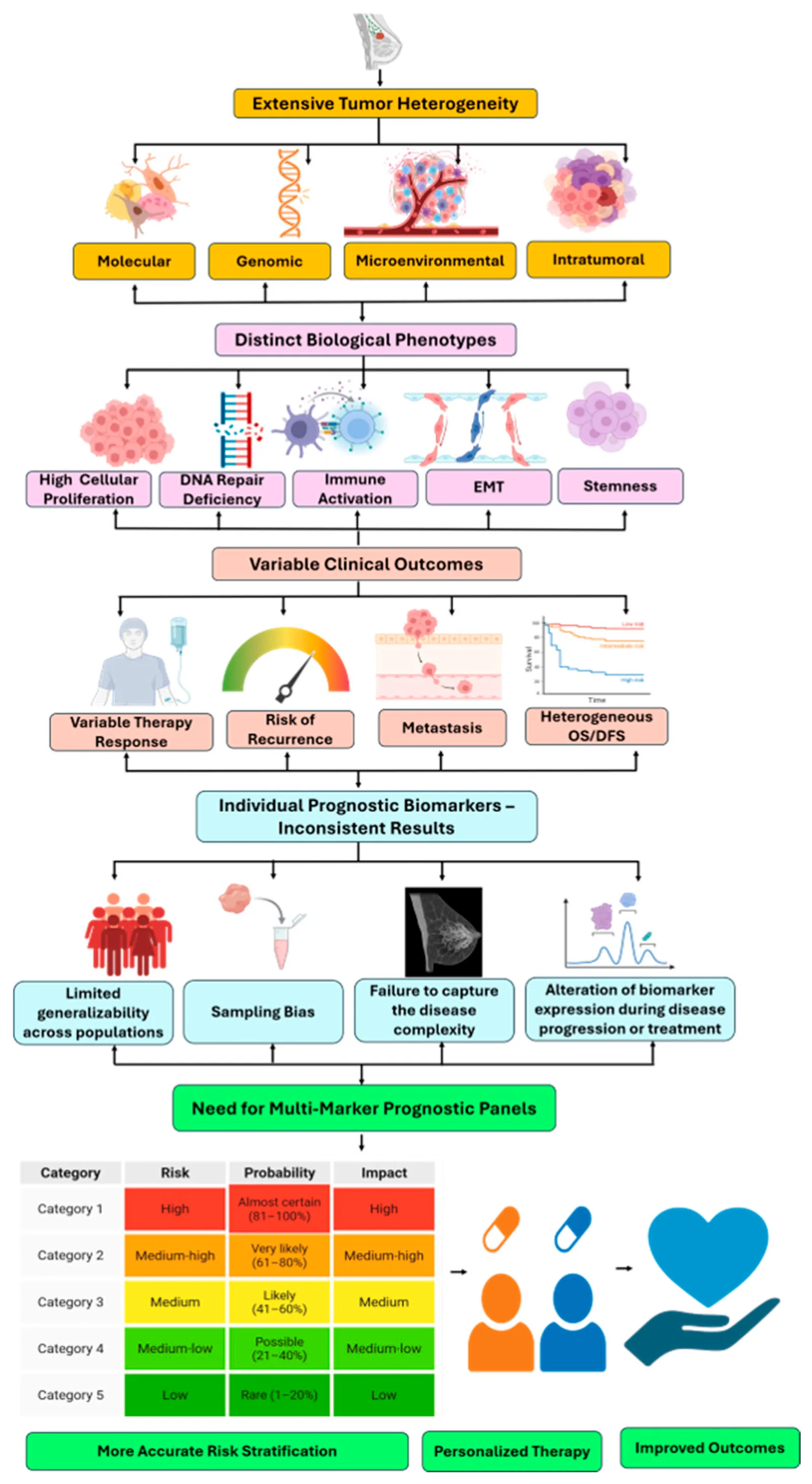
Impact of heterogeneity on prognosis and reduced reliability of individual markers in TNBC.

Figure 2 depicts how the heterogeneity of TNBC at multiple biological levels significantly influences clinical outcomes, emphasizing the dire need for development of multi-marker panels for efficient prognostication.

## 6. Multi-Marker Panels and Prognostic Signatures in TNBC

As discussed previously, the prognostic value of individual biomarkers is significantly limited by the complex molecular heterogeneity of TNBC. Consequently, to overcome the incomplete picture offered by individual biomarkers, researchers are rapidly moving toward multi-marker panels and multi-gene signatures for TNBC prognostication. These modern approaches view TNBC as a continuously evolving ecosystem and thereby can provide a more comprehensive representation of tumor biology. A multi-marker panel refers to a physical group of biomarkers or targets being tested simultaneously, eliminating the need to run separate individual tests. A multi-gene signature, on the other hand, is a statistical algorithm derived from the multi-marker panel that helps to predict the progression of a patient’s disease.

As far as TNBC is concerned, multi-marker panels consider its spatial and temporal heterogeneity via targeting several phenotypic pathways involving cellular proliferation, immune regulation, and EMT. As a result, they do not focus on a single checkpoint but instead evaluate an entire cluster of points associated with the disease, thereby capturing the overall pattern. This mitigation of tumoral heterogeneity reduces the risk of false negatives/positives and, in turn, improves prognostic accuracy. Second, multi-marker panels capture not only the aggressive nature of cancer cells but can also potentially measure to what extent the patient’s immune system is combating the cancer cells. Thus, by weighing both sides, these panels provide a complete picture and help to maintain the net balance. Additionally, single biomarkers often tend to place rigid cut-offs on a patient’s disease condition and categorize them simply into “positive” and “negative” groups. However, multi-marker panels do not only rely on “yes” or “no.” Instead, they aim to provide a continuous biological scaling or a personalized spectrum for a patient by combining all of the available data, ultimately allowing for better stratification of patients into low-, intermediate-, and high-risk groups.

### 6.1. Gene Expression-Based Prognostic Signatures

Similar to individual biomarkers, multi-marker panels and prognostic signatures can also be classified into different categories based on the molecular target. Among these, gene expression-based prognostic signatures have emerged as powerful tools for risk stratification in TNBC. It is a well-known fact that in most tumors, including TNBC, several biochemical processes occur simultaneously and biological pathways interact dynamically. Gene expression-based signatures can help to capture these pathway-level alterations and consequently provide better prognostic insights. These signatures combine the expression patterns of multiple genes, enabling an accurate classification of TNBC patients based on their survival outcomes and risk of recurrence. Importantly, gene expression signatures mainly include genes associated with cellular progression and proliferation, DNA damage response, immune regulation, EMT, and metabolic reprogramming.

Table 4 provides detailed insights into the different classes of gene expression signatures along with their prognostic significance.

### 6.2. Protein-Based Composite Panels

Protein-based composite panels constitute an important class of multi-marker prognostic models for TNBC. In comparison to individual protein markers that reflect only a single biological process, protein-based composite panels consider multiple proteins associated with complementary biological pathways [79]. This integration helps to capture the multifaceted nature of TNBC, particularly the complex crosstalk between the tumor cell and the tumor microenvironment, thereby providing a more thorough evaluation of overall tumor behavior. Protein-based composite panels typically incorporate protein biomarkers associated with:

✓ Cellular proliferation—e.g., Ki-67, cyclin B1, cyclin D1;
✓ DNA damage response—e.g., p53, BRCA1, BRCA2, PARP1;
✓ Growth factor signaling—e.g., EGFR, insulin-like growth factor 1 receptor (IGF-1R);
✓ EMT and Invasion—e.g., E-cadherin, N-cadherin, vimentin, matrix metalloproteinase-2 (MMP-2), matrix metalloproteinase-9 (MMP-9);
✓ Immune activation—e.g., PD-L1, CD4, CD8, CD68;
✓ Angiogenesis—e.g., VEGF, HIF-1α.

Numerous studies have assessed different combinations of protein biomarkers to improve prognostic accuracy in TNBC. One of the most studied protein biomarker combinations is that of Ki-67 alongside p53. This combination effectively integrates the proliferative activity of Ki-67 with the genomic instability associated with p53. Tumors having elevated Ki-67 expression in conjunction with aberrant p53 expression generally have a higher histological grade and poorer OS and DFS compared to tumors wherein only one of the biomarkers is abnormally expressed. Moreover, studies have also added EGFR to the combination of Ki-67 and p53 for better characterization of the aggressive tumor biology of TNBC [12,79]. Furthermore, panels comprising immune system-related proteins are also emerging. One of the strongest panel systems consists of PD-L1, CD8, and stromal TILs. Increased levels of CD8 together with abundant stromal TILs and PD-L1 expression have been associated with favorable prognosis in TNBC [79]. Similarly, it was also found that the expression of epithelial human leukocyte antigen-DR, stromal CD4, and stromal inducible T-cell costimulator was significantly higher in TNBC patients with long-term DFS in comparison to those patients in which the disease had relapsed [127]. A study by Bottai et al. revealed that the presence of PD-1 and lymphocyte activation gene 3 (LAG-3) proteins may potentially be associated with a positive prognosis rate in TNBC patients [128].

One of the major advantages that protein-based composite panels offer is their high compatibility with routinely used clinical pathology techniques such as immunohistochemistry (IHC). Consequently, these panel systems can be readily applied to formalin-fixed paraffin-embedded tissue sections, making them not only cost-effective but also easier to implement than other molecular assays. Lastly, their overall reproducibility and ability to facilitate personalized treatment make them tools with great translational potential.

### 6.3. Immune Prognostic Scores

TNBC is widely recognized as the most immunogenic subtype of breast cancer due to its significant genomic instability, severe tumor mutational and neoantigen burden, and elevated levels of infiltrating immune cells. Accumulating scientific evidence has suggested that the different parameters associated with immune cells in terms of their number, functional status, and composition have a profound effect on TNBC progression [129]. Immune cells work in tandem with other immunological components, such as cytokines, chemokines, and stromal elements, to elicit a suitable immune response. Individual biomarkers often reflect only one aspect of this response. For example, CD8 T-cells on their own indicate the cytotoxic or killing mechanism of the immune system but only offer minimal insights into immunosuppressive processes. On the other hand, immune scores evaluate multiple immune parameters simultaneously, depicting a more accurate picture of the immune dynamics within the TNBC.

Most immune prognostic models integrate biomarkers from multiple categories, such as:

✓ TILs;
✓ Cytotoxic T-cell markers—e.g., CD8, granzyme B, perforin;
✓ Immune checkpoint proteins—e.g., PD-1, PD-L1, LAG-3, T-cell immunoglobulin and mucin-domain containing protein 3 (TIM-3);
✓ Macrophage polarization markers—e.g., CD68, CD206, CD163;
✓ Cytokines and chemokines—e.g., interferon-gamma, IL-6, IL-8, CXCL9, CXCL10.

Galon et al., in their foundational review, extensively highlighted the concept of the “Immunoscore”, which uses an individual’s immune profile as a parameter for classifying cancer and as an indicator of prognosis [130]. Immune scoring reflects the fact that the host’s immune system primarily regulates the development of cancer, thereby underscoring the importance of immunological biomarkers for prognostication. It is a strong prognostic indicator for OS and DFS, especially in the early stages of cancer, where higher Immunoscore values imply robust anti-tumor immunity. The Immunoscore is based on the density and spatial distribution of memory (CD3^+^) and cytotoxic (CD8^+^) T-lymphocytes in the center of the tumoral zone and at the invasive margin. It was originally developed for colorectal cancer but now has also been adapted for breast cancer research. However, no standardized TNBC-specific Immunoscore has yet been approved for routine clinical use.

Araujo et al. developed and evaluated the prognostic value of a 3-gene score in TNBC patients with different molecular subtypes [131]. The three genes included in the study were *CCL5*, *DDIT4*, and *POLR1C*. Post analysis, their findings suggested that the 3-gene score could predict the risk of distant recurrence in TNBC patients, particularly in those with IM and MSL subtypes. Interestingly, it was also revealed that a lower TNBC 3-gene score was associated with an increase in the number of TILs, implying a potential correlation between the two parameters. Similarly, in another study, the expression of five immune biomarkers, viz. CD3^+^, CD4^+^, CD8^+^, CD19^+^, and CD163^+^ (representing the major classes of immune cells), was assessed for its prognostic role in TNBC using an automated approach for calculating the Immunoscore [132]. Choi et al. further expanded the panel of immune biomarkers and developed a novel combined immune score-based model comprising eight immune markers (PD-1, PD-L1, PD-L2, IDO, TIM3, OX40, OX40L, and H7-H2) for evaluating prognosis in TNBC [133]. The expression of these eight immune targets was assessed using IHC in tumor cells as well as immune cells, based on which TNBC patients were classified into high- and low-CIS groups, respectively, with a higher score associated with favorable rates of PFS and OS. A prognostic signature consisting of twelve immune-related genes—*TDO2*, *CHIT1*, *CARML2*, *HLA-C*, *ADIRF*, *C19orf33*, *CA8*, *AHNAK2*, *RHOV*, *OPLAH*, *THEM6*, and *NEBL*—has also been developed, wherein *TDO2*, *CHIT1*, *CARMIL2*, and *HLA-C* have been associated with favorable prognosis, while *ADIRF*, *C19orf33*, *CA8*, *AHNAK2*, *RHOV*, *OPLAH*, *THEM6*, and *NEBL* contribute to a dismal rate of prognosis [134]. Notably, the signature of these twelve genes, which are associated with the expression of immune checkpoints, has the potential to aid in the choice of immunotherapy for TNBC.

### 6.4. Multi-Omic Prognostic Models

Multi-omic prognostic models are the most recent generation of multi-marker panels being employed for prognostic stratification in TNBC. Progression and development of any tumor is a result of several coordinated alterations occurring at various molecular levels. Multi-omic prognostic models assess these interconnected molecular events simultaneously and collate the information obtained to potentially capture the cause and subsequent phenotypic consequences associated with tumor progression. This integration of different datasets offers a comprehensive approach toward tumor prognosis in terms of metastasis, recurrence, and survival.

The components of multi-omic prognostic panels commonly include:

✓ Genomics—For copy number variation analysis, HR deficiency (e.g., *TP53*, *PTEN*, *RB1*);
✓ Transcriptomics—For evaluation of mRNA expression profiles of genes associated with proliferation, DNA repair, immune activation, and EMT (e.g., *MKI67*, *AURKA*, *CD8*);
✓ Epigenomics—For identification of regulatory mechanisms influencing gene expression without alteration of the DNA sequence (e.g., DNA methylation analysis, histone modification patterns);
✓ Proteomics—For interpretation of protein abundance (e.g., EGFR, PARP1, PD-L1);
✓ Metabolomics—For quantification of metabolites reflecting altered metabolism (e.g., glycolysis, oxidative phosphorylation, lipid metabolism).

Interestingly, prognostic models involving the use of artificial intelligence and machine learning techniques, viz. Random Forest, deep learning, and neural networks, have been developed for prognostication. Le et al. developed an automated prognostic model for the detection of invasive breast cancer using convolutional neural networks analysis [135]. This model considered the structural patterns and overall spatial distributions of TILs for risk stratification in invasive breast cancer. Consequently, Wang et al. developed the first transcriptome-wide expression–morphology analysis model where deep convolutional neural networks were utilized to elucidate the mRNA expression patterns and associated proliferation markers in breast cancer [136].

Moreover, several studies have combined copy number alterations, somatic mutation profiles, RNA sequencing, and DNA methylation analysis to develop integrated models using data obtained from The Cancer Genome Atlas (TCGA). Lehmann et al. carried out a thorough integrative analysis pertaining to TNBC subtypes wherein the copy number, mutation, gene expression, DNA methylation, and proteomic data of TNBC patients listed in TCGA and the Clinical Proteomic Tumor Analysis Consortium were comprehensively analyzed [137]. This study provided detailed insights into the complex biology of TNBC subtypes and highlighted potential approaches for the treatment of TNBC. Chai et al., on the other hand, used an integrated multi-omic approach combining single-cell RNA sequencing, immune infiltration analysis, and in vitro functional assays to develop an immune-related four-gene prognostic signature for predicting outcomes in TNBC patients [138].

**Figure 3:**
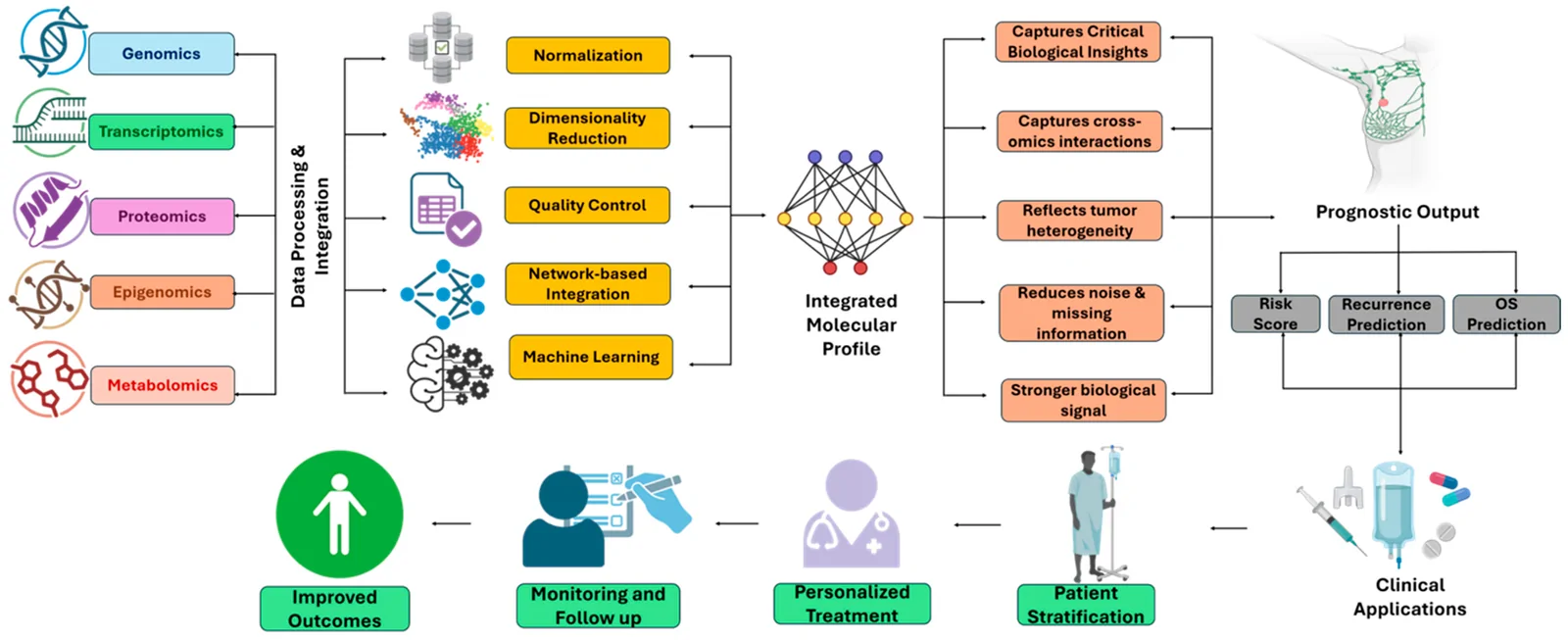
Multi-omic integration framework for prognostic assessment in TNBC.

Figure 3 illustrates how the development of an integrated framework involving multi-omic data can potentially lead to better outcomes for TNBC patients.

## 7. Clinical Translation of Multi-Marker Panels: Challenges and Future Perspectives

Despite significant advancements in multi-marker panels and prognostic signatures, their translation into routine clinical practice, particularly for TNBC, remains quite challenging. Several biological, technical, and clinical constraints hinder the widespread implementation of these model systems. First and foremost is the complex heterogeneous nature of TNBC. The remarkable heterogeneity of TNBC at the genomic, transcriptomic, immune, and proteomic levels substantially impedes the prognostic accuracy of multi-marker panels across its different subtypes [115]. For example, a prognostic panel may work for the LAR subtype but may end up showing poor results for the basal or mesenchymal subtypes. Second, as mentioned previously, due to the extensive intra-tumoral heterogeneity associated with TNBC tumors, a single tissue biopsy may not be able to accurately reflect the state of the entire tumor ecosystem. As a result, different regions of the tumor may express different stromal composition, mutations, immune cell infiltration, and EMT status, leading to uncertainty in terms of prognostication.

Another critical drawback associated with multi-marker panels is the lack of standardization across different study platforms. Some studies use RNA sequencing and microarrays, some employ IHC, while others use proteomic-based approaches and DNA methylation arrays. Hence, direct comparison of prognostic risk scores between these platforms is not possible. In addition, there is no universal agreement established for risk stratification thresholds, scoring criteria, or cut-offs. Different researchers use different thresholds, further leading to limited reproducibility across studies [139]. Importantly, although several prognostic TNBC models have been reported, most of them have been derived from retrospective datasets such as TCGA or the Molecular Taxonomy of Breast Cancer International Consortium, and they are not validated in large, prospective, independent cohorts. The lack of such rigorous validation makes it difficult to routinely implement these models into clinical practice and raises concerns about their generalizability and overall robustness [140].

Moreover, most prognostic signatures are generated from a single pre-treatment sample. However, TNBC tumors continuously evolve in response to chemotherapy, immunotherapy, and metastasis. Hence, prognostic signatures often fail to consider these dynamic changes occurring during tumor progression. Furthermore, a static biomarker panel may not be able to detect the development of therapy-resistant clones, resulting in poor prediction of long-term outcomes [139,140]. Moreover, multi-omic approaches require specialized laboratory infrastructure and expertise in the field of bioinformatics, making it particularly expensive. Many low- and middle-income countries, where TNBC is highly prevalent, may lack the necessary resources to perform such comprehensive molecular profiling. Finally, despite the edge that multi-marker panels offer over individual biomarkers, many researchers still prefer to focus on only one biological process (e.g., proliferation) at a time. As a result, despite their considerable biological promise for improving prognostic stratification in TNBC, multi-marker panels remain primarily investigational in nature. A rigorous validation—externally as well as prospectively—is required before they can be incorporated routinely into clinical decision-making. Thus, establishment of the routine clinical utility of these models serves as an important direction for future research.

Nonetheless, researchers need to move beyond using individual biomarkers toward multi-marker approaches wherein clinicopathological factors, genomic markers, transcriptomic markers, epigenetic markers, protein-based markers, immune-related markers, and liquid biopsy markers are combined and analyzed simultaneously. The effective implementation of multi-marker panels in TNBC will require the standardization of reporting guidelines and scoring methods, prospective multicenter validation, data integration, and development of cost-effective assays to achieve their full potential in precision oncology [141].

## 8. Clinical Relevance of TNBC Prognostic Biomarkers in the Contemporary Treatment Landscape

It is critical to emphasize that the clinical assessment of these prognostic biomarkers in TNBC should be increasingly evaluated with respect to contemporary systemic treatments. The prognostic value of a biomarker may be influenced by the treatment administered. Moreover, as discussed previously, certain biomarkers provide essential predictive insights, benefiting the overall treatment. For example, TILs have shown substantial prognostic evidence in early TNBC, with their high levels corresponding to better survival outcomes. However, TILs are also being investigated for their potential to predict the response to immunotherapies involving the use of immune checkpoint inhibitors [142]. Similarly, PD-L1 has been used to identify patients eligible for specific treatment settings. BRCA1/2 alterations also help to pinpoint patients qualifying for treatment with PARP inhibitors [2]. Therefore, the clinical relevance of TNBC biomarkers should be evaluated not only by their correlation with survival outcomes but also in the context of treatment selection and how treatment approaches alter their prognostic significance.

## 9. Conclusions

TNBC continues to remain one of the most clinically challenging subtypes of breast cancer owing to its diverse molecular nature, aggressive biological behavior, high rate of metastases, and limited therapeutic alternatives. Accurate prognostic stratification is therefore the key not only for identifying patients at greater risk of recurrence but also for guiding their treatment and developing new therapies. Over the past decade, significant progress has been made in terms of identifying potential individual prognostic biomarkers and classifying them into relevant groups based on their molecular features. Collectively, all of these individual prognostic biomarker classes (e.g., clinicopathological factors, genomic markers, transcriptomic markers, epigenetic markers, protein-based markers, immune and tumor microenvironment-related markers, circulating and liquid biopsy-based markers) have considerably improved our understanding of TNBC biology. However, they fail to effectively represent the overall disease behavior, driving increasing interest toward multi-marker panels. These prognostic panels, comprising mainly gene expression signatures, protein-based composite panels, immune prognostic scores, and multi-omic models, can help to simultaneously capture the multiple hallmarks associated with tumor progression. Despite these encouraging developments, several critical obstacles persist, hindering their implementation for routine clinical prognosis of TNBC. Therefore, the future of prognostic assessment in TNBC depends on the successful translation of these multi-marker panels into routine management through rigorous clinical validation, thereby facilitating personalized treatment approaches.

## Figures and Tables

**Table 1:** Overview of the key molecular characteristics of TNBC molecular subtypes.

	TNBC Molecular Subtype					
	BL1	BL2	IM	M	MSL	LAR
Key Molecular Characteristics	Enhanced cellular proliferation [12]	Exhibition of growth factor signaling pathways, e.g., epidermal growth factor signaling [5]	Involvement of immune cell responses, viz. antigen presentation and processing, immune cell signaling, and cytokine signaling [12]	Exhibition of growth factor pathways and epithelial–mesenchymal transition	Exhibition of growth factor pathways and epithelial–mesenchymal transition	Enhanced expression of androgen receptor [13]
	Loss of cell cycle regulation [12]	Involvement of glycolysis and gluconeogenesis pathways [12]	Dysregulation of key signal transduction pathways, including TNF-α, JAK/STAT, and NF-κB [11]	Abnormal regulation of WNT, ALK, and TGF-β signaling pathways [11]	Extremely susceptible to mTOR inhibitors and tyrosine kinase inhibitors [12]	Highly responsive to androgen receptor antagonists, e.g., Biclutamide [12]
	Activation of DNA damage response pathways	Increased expression of epidermal growth factor receptor, p63, and CD10 [11]			Aberrant regulation of Rho, ERK1/2, EGFR, PDGF, and PI3K [11]	Associated with lower disease-free survival and overall survival
	Elevated expression of Ki67 mRNA [12]					
	Sensitive to platinum-based chemotherapies [14]					

**Table 2:** Classification of TNBC individual prognostic biomarkers on the basis of clinical implementation.

Clinical Status	Category	TNBC Prognostic Biomarker	References
Clinically established	Clinicopathological	Tumor size	[74,115,117]
		Histological grade	
		Age	
		Lymph node involvement	
	Immune and tumor microenvironment	TILs	
Clinically relevant but context-dependent	Clinicopathological	Histological tumor subtype	[74,118]
	Genomic and transcriptomic	*BRCA1/2*	
	Protein	PD-L1 Ki67	
Emerging	Genomic and transcriptomic	p53	[74,79]
		ABCC9	
		NKAPL	
		miRNAs	
		lncRNAs	
		circRNAs	
	Epigenetic	DNA methylation signatures	
		Histone post-translational modification signatures	
	Protein	AR	
		EGFR	
		VEGF	
		E-cadherin	
		Aquaporins	
	Immune and tumor microenvironment	*IL18R1*	
		ITPKC	
	Circulating and liquid biopsy	CTCs	
		ctDNA	
		Extracellular vesicles	

**Table 3:** Limitations of individual prognostic biomarkers for assessment in TNBC.

TNBC Prognostic Biomarker Category	Major Limitations	References
Clinicopathological factors	Limited precision Poor personalized risk stratification	[12,79]
Genomic and transcriptomic markers	High variability across patient cohorts Batch-to-batch variations Platform dependency	[12,79,119]
Epigenetic markers	Highly dynamic in nature Lack of standardization Assay complexity	[120]
Protein-based markers	Subjective scoring and cut-off inconsistency Intra-tumoral heterogeneity Reproducibility concerns	[12,79]
Immune and tumor microenvironment-related markers	Spatial heterogeneity Dynamic immune changes Context-dependent interpretation	[121,122]
Circulating and liquid biopsy-based markers	Sensitivity issues Standardization challenges High cost	[79,123]

**Table 4:** Different classes of gene expression-based prognostic signatures.

Gene Expression Signature Type	Features	Key Genes Included	Prognostic Significance	References
Proliferation-associated signatures	Extensively studied in TNBC Reflects tumor growth dynamics and cell cycle dysregulation (defining hallmarks of TNBC) Tumors with an elevated expression of proliferation genes typically exhibit: - Rapid tumor growth - Higher histological grade - Enhanced metastatic potential - Lower recurrence-free survival	MKI67—Encodes the widely used proliferation marker protein Ki-67 CCNB1 (Cyclin B1)—Mediates the G2/M transition by activating cyclin-dependent kinase 1 (CDK1) AURKA (Aurora Kinase A)—Responsible for centrosome maturation and spindle assembly during mitosis PLK1 (Polo-like Kinase 1)—Essential for spindle assembly, entry into mitosis, and cytokinesis	TNBC tumors displaying higher proliferation signatures frequently fall under basal-like subtypes, viz. BL1 (due to abundance of cell cycle associated networks) High proliferation scores generally correspond with a reduction in OS, DFS, and an increase in the relapse rate Tumors with elevated proliferation scores may have greater sensitivity to conventional chemotherapy, thereby initiating a better initial therapeutic response (a prognostic paradox) Proliferation genes are often a part of well-established multi-gene signatures used for breast cancer prognostication such as Oncotype DX and MammaPrint (used primarily in hormone receptor-positive, HER2-negative breast cancers)	[10,11,79,124]
DNA repair deficiency signatures	Helps to evaluate the functionality of DNA damage response cascades, viz. homologous recombination (HR) A significant percentage of TNBC tumors exhibit abnormalities in DNA repair pathways, leading to accumulation of mutations, genomic instability, and aggressiveness Tumors such as breast, prostate, and ovarian cancer exhibit “BRCAness”—a phenomenon wherein the tumors without BRCA1 and BRCA2 mutations display molecular aberrations similar to those having these mutations due to defects in the HR repair mechanism	BRCA1 & BRCA2—Critical genes associated with HR repair RAD51—Plays a role in strand invasion during HR repair ATM (Ataxia-Telangiectasia Mutated Kinase)—Senses DNA damage, particularly DNA double-stranded breaks and coordinates their repair ATR (ATM and Rad3-related Kinase)—Maintains genome stability during DNA replication CHEK1 (Checkpoint Kinase 1) & CHEK2 (Checkpoint Kinase 2)—Essential for DNA repair and cell cycle arrest	TNBC tumors with abnormalities in HR are typically associated with: - Higher histological grade - Pronounced genomic instability - Higher metastatic potential - Greater recurrence risk Interestingly, these tumors also tend to have a higher susceptibility to platinum-based chemotherapies and poly (ADP-ribose) polymerase (PARP) inhibitors (another prognostic paradox) Recently, numerous HR deficiency (HRD) scoring systems have been developed to accurately measure the dysregulation of DNA repair. These systems combine multiple genomic features, such as loss of heterozygosity (LOH) and telomeric allelic imbalance (TAI), to improve the overall prognostic stratification	[79,115,125,126]
EMT and metastasis-associated signatures	Tumor growth, metastatic spread, therapeutic resistance, and disease recurrence in TNBC are all known to be significantly influenced by EMT These gene expression signatures have emerged as valuable tools for assessing the poor clinical outcomes and TNBC aggressiveness in patients	CDH1 (E-cadherin)—Key protein mainly responsible for cell–cell adhesion VIM (Vimentin)—Intermediate filament protein commonly associated with metastasis SNAI1 (Snail) & SNAI2 (Slug)—Master EMT transcription factors that drive invasion and metastasis TWIST1—An EMT regulator that plays a crucial role in metastasis	EMT is particularly prevalent in M and MSL molecular subtypes of TNBC Elevated expression of EMT-related genes is frequently linked to - Lower OS - Reduced DFS - Stronger risk of metastasis - Greater likelihood of recurrence Tumors with high EMT signature scores generally have a higher chance of developing distant metastases, especially to the brain, lungs, and liver—all of which are prominent metastatic sites in TNBC Importantly, ineffective responses to neoadjuvant chemotherapy (NACT) are observed in TNBC patients having higher EMT signature scores Combination of EMT-related markers along with stemness/immune signatures offer better prognostic outcomes compared to either signature alone	[11,79,115]

## Data Availability

No new data were created or analyzed in this study. Data sharing is not applicable to this article.

## Abbreviations

The following abbreviations are used in this manuscript:

TNBC	Triple-negative breast cancer
ER	Estrogen receptor
PR	Progesterone receptor
HER2	Human epithelial growth factor receptor 2
PARP	Poly ADP ribose polymerase
BL1	Basal-like 1
BL2	Basal-like 2
IM	Immunomodulatory
M	Mesenchymal
MSL	Mesenchymal stem-like
LAR	Luminal androgen receptor
EMT	Epithelial–mesenchymal transition
OS	Overall survival
DFS	Disease-free survival
ABCC9	ATP-binding cassette subfamily C member 9
NKAPL	NF-Kβ-activating protein-;ike
MiRNA	MicroRNA
ARRB1	Arrestin beta 1
lncRNA	Long non-coding RNA
DNMT3A	DNA methyltransferase 3α
EZH2	Enhancer of zeste homolog 2
PKM2	Pyruvate kinase M2
EGFR	Epidermal growth factor receptor
VEGF	Vascular endothelial growth factor
PD-L1	Programmed death ligand-1
TIL	Tumor infiltrating lymphocyte
ITPKC	Inositol 1,4,5-trisphosphate 3-kinase C
CTC	Circulating tumor cell
cfDNA	Circulating cell-free DNA
ctDNA	Circulating tumor DNA
EVs	Extracellular vesicles
IGF-IR	Insulin-like growth factor 1 receptor (IGF-IR)
MMP-2	Matrix metalloproteinase-2
MMP-9	Matrix metalloproteinase-9
LAG-3	Lymphocyte activation gene 3
IHC	Immunohistochemistry
TIM-3	T-cell immunoglobulin and mucin-domain containing protein 3
TCGA	The Cancer Genome Atlas
